# Yersinia virulence factors - a sophisticated arsenal for combating host defences

**DOI:** 10.12688/f1000research.8466.1

**Published:** 2016-06-14

**Authors:** Steve Atkinson, Paul Williams

**Affiliations:** 1 Centre for Biomolecular Sciences, School of Life Sciences, University of Nottingham, Nottingham, UK

**Keywords:** Yersinia pestis, yersiniae, ail locus, pH6 antigen, virulence factors

## Abstract

The human pathogens *Yersinia pseudotuberculosis* and *Yersinia enterocolitica* cause enterocolitis, while *Yersinia pestis* is responsible for pneumonic, bubonic, and septicaemic plague. All three share an infection strategy that relies on a virulence factor arsenal to enable them to enter, adhere to, and colonise the host while evading host defences to avoid untimely clearance. Their arsenal includes a number of adhesins that allow the invading pathogens to establish a foothold in the host and to adhere to specific tissues later during infection. When the host innate immune system has been activated, all three pathogens produce a structure analogous to a hypodermic needle. In conjunction with the translocon, which forms a pore in the host membrane, the channel that is formed enables the transfer of six ‘effector’ proteins into the host cell cytoplasm. These proteins mimic host cell proteins but are more efficient than their native counterparts at modifying the host cell cytoskeleton, triggering the host cell suicide response. Such a sophisticated arsenal ensures that yersiniae maintain the upper hand despite the best efforts of the host to counteract the infecting pathogen.

## Introduction

Across an infection timeline, the host and invading bacterial pathogen each vie for supremacy. At any given time, either may have the upper hand, but the final outcome of this battle ultimately determines the fate of the host. The triggered host response will aim to reduce the infectivity of the pathogen, but in order to stay one step ahead many bacterial species have evolved sophisticated strategies to ensure they can successfully cause infection following colonisation.

The three human pathogens belonging to the genus Yersinia employ a range of virulence factors that confer efficient adherence to host cells/tissues and subvert host cell functions. This mini-review highlights the key virulence factors that constitute the virulence arsenal of Yersinia spp. and how such a sophisticated suite of biological weapons enables these pathogens to combat host defences.

*Yersinia pseudotuberculosis*, *Yersinia pestis*, and *Yersinia enterocolitica* are highly adaptable psychrotrophic primary human pathogens. *Y. pseudotuberculosis* and *Y. enterocolitica* cause self-limiting gastric infections. *Y. pestis* is a recently evolved near-identical subclone of *Y. pseudotuberculosis* ^1, 2^ with approximately 98% identity at the DNA level. Its strategy for transmission relies on the colonisation of rat fleas, which then carry *Y. pestis* between the rodent host and humans ^3^. Once inside the human host, *Y. pestis* can cause bubonic, pneumonic, and septicaemic plague with mortality rates approaching 100% without antibiotic treatment ^4^. The World Health Organisation considers *Y. pestis* a ‘re-emerging’ pathogen that, worryingly, is capable of acquiring resistance to multiple antibiotics ^5^ and is also a serious potential bioterrorism threat. The differences in lifestyle and virulence between *Y. pseudotuberculosis* and *Y. pestis* are mostly attributable to minor genomic differences on the respective chromosomes and the presence of two additional virulence plasmids that *Y. pestis* possesses.

## The Yersinia type three secretion system

**Figure 1.**
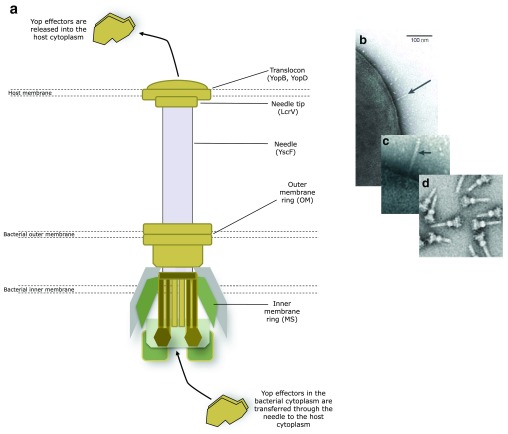
Assembly of the type three secretion system (T3SS) needle. The needle is fixed into the bacterial inner and outer membrane and protrudes from the surface to penetrate the host membrane. The translocon forms a channel through the host membrane and the Yop effectors are transferred into the host from the bacterial cytoplasm via the needle and translocon ( **a**). The needle protrudes from the bacterial surface prior to host cell penetration ( **b**, **c** arrowed). *Salmonella typhimurium* T3SS needles isolated from the bacterial membrane ( **d**). ( **a**) adapted from 133, ( **b**) reproduced with permission and taken from reference 56, ( **c**) reproduced with permission and taken from reference 16, and ( **d**) reproduced from reference 134.

The key Yersinia virulence determinants and certainly the most comprehensively studied are those secreted via a type three secretion system (T3SS). To evade host innate immunity and to enable the pathogen to replicate and propagate extracellularly, all human pathogenic Yersinia species harbour an approximately 70 kb virulence plasmid. Located on this plasmid is a set of genes whose transcription is activated by temperatures of 37 ^°^C in the presence of millimolar concentrations of calcium, conditions representing the mammalian host. These genes code for the T3SS ‘nanomachine’, a hypodermic needle-like structure (the injectisome) and the translocon, which forms a pore across the host cell membrane ( Figure 1). Along with a combination of regulators and chaperones, the T3SS’s primary function is to inject multiple toxic Yersinia effector proteins (Yops) directly into the eukaryotic host cell cytosol. Once inside, they subvert host cell signalling pathways and trigger a pre-programmed metabolic chain reaction that results in apoptosis ^6, 7^. Yops also inhibit phagocytosis and block cytokine production.

## The structure of the T3SS needle and translocon

Structurally, the base of the injectisome is composed of a number of proteins that adopt a cylindrical architecture similar to that of the flagellar basal body ^8^ that are directed to the membrane by the secretion (Sec)-dependent pathway ^9^. The injectisome incorporates two membrane rings termed the MS (membrane and supramembrane) and OM (outer membrane) rings. These are connected to five integral membrane proteins that play a role in exporting proteins ^10, 11^ ( Figure 1). The export apparatus itself is flanked by YscQ, which facilitates the binding of the ATPase YscN and the secretion substrate-chaperone complexes ^12^. YscN provides the proton motive force necessary for driving the secretion of the Yop effectors ^9, 13, 14^.

**Figure 2.**
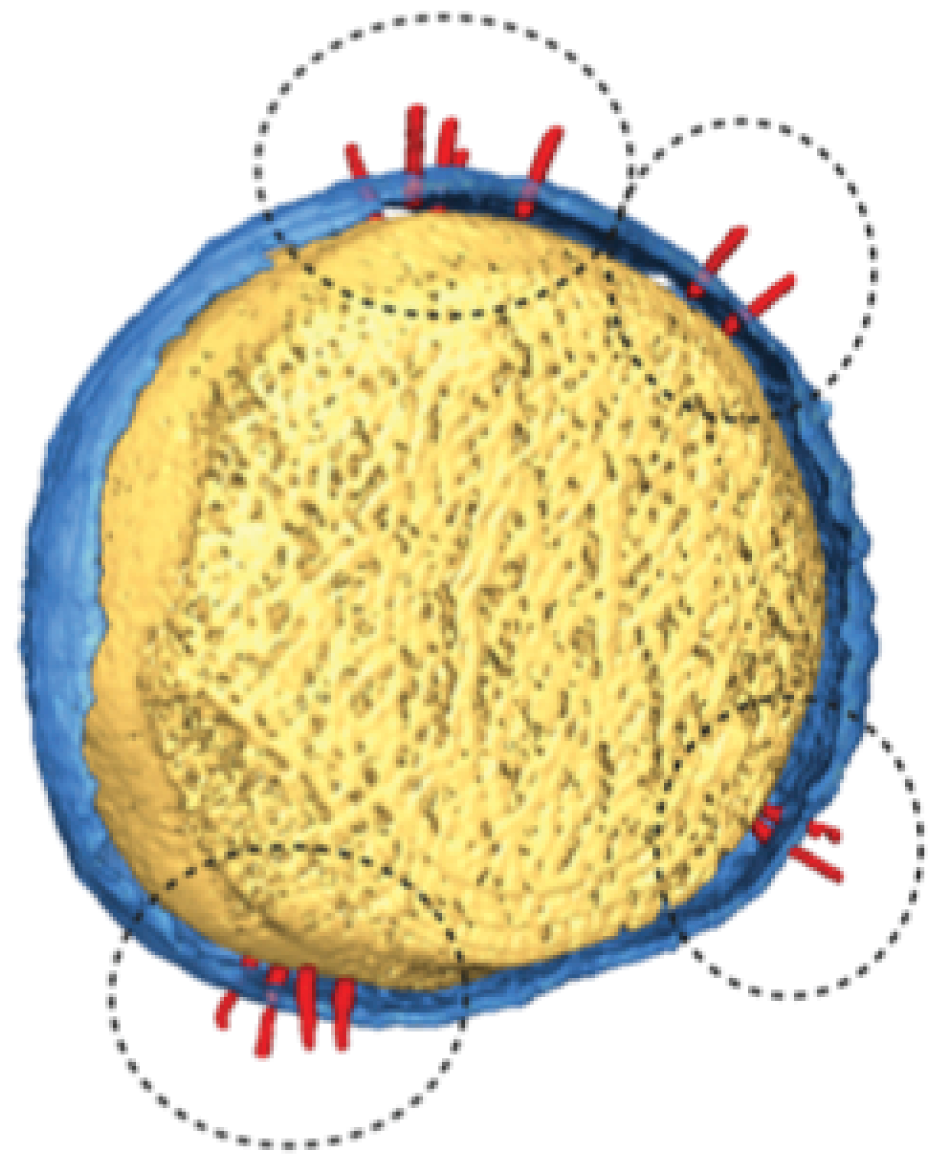
Type 3 secretion system (T3SS) needles (circled) appear to cluster together as they form at the cell surface. Reproduced from reference 20.

Protruding into the extracellular space from the basal body is a hollow needle formed by the helical polymerisation of YscF protein subunits ^9, 15, 16^. YscF is exported and polymerised in a T3SS-dependent manner along with YscP, a protein akin to a molecular ruler that determines the length of the needle and limits its size ^17– 19^. It has recently been shown that fully formed T3SS needles form clusters on the bacterial cell surface and new needles appear to localise to these clusters rather than being randomly distributed ^20^ ( Figure 2). The needle tip is capped with LcrV ^21, 22^, a protein that directs the formation of a pore or ‘translocon’ ^23^. The translocon consists of a tripartite protein pore, which is inserted into host cell membranes and drives the translocation of Yop effectors into the host target cell cytoplasm. The pore is composed of the transmembrane proteins YopB and YopD ^23^ and the injectisome tip complex LcrV ^24– 26^. Bacteria lacking the tip and translocon proteins are able to secrete effectors into the extracellular environment but are defective in translocating Yops into host cells ^27– 29^.

## Chaperones facilitate the formation and operation of the T3SS

Given the complexity of the T3SS, part of its sophistication relates to its in-built ability to discriminate between structural and secretion substrates, providing strict order to ensure the needle is assembled and polymerised before translocon and Yop effector secretion ^30^. Such ordering requires specific chaperones, typically small protein dimers that protect the target T3SS protein from degradation ^31, 32^ and prevent premature oligomerisation ^24^ and also ushering into the injectisome. These T3SS chaperones are usually subdivided into three classes: class I chaperones bind the Yop effector proteins and often share high structural conservation, class II chaperones associate with the translocon proteins YopB, YopD, and LcrV, and class III chaperones tend to form heterodimers and associate with structural components of the injectisome.

## The Yop effectors

The Yop effector proteins are virulence factors synthesised in the bacterial cytoplasm and secreted through the T3SS needle and translocon into eukaryotic target cells ( Figure 1). Four of these (YopE, YopT, YpkA, and YopH) are involved in disrupting the normal activities of the cytoskeleton and, apart from YopH, also target an important group of eukaryotic cell signalling components, the RhoA family of small GTPases that direct cytoskeletal rearrangements necessary for phagocytosis. YopE is a functional mimic of eukaryotic GTPase-activating proteins (GAPs) ^33^ and disrupts the actin cytoskeleton ^34– 36^, resulting in the inhibition of phagocytosis by macrophages. YopT suppresses RhoA-mediated signalling by cleaving the post-translationally modified Rho GTPases ^37^, which ultimately prevents the formation of the phagocytic cup for bacterial internalisation, and inhibits the assembly of focal adhesion complexes required for the development of pseudopodia and macrophage migration ^38, 39^. YpkA (YopO in *Y. enterocolitica*) associates with RhoA family proteins ^40, 41^ and inhibits phagocytosis ^42, 43^ by binding to and phosphorylating actin that is used as bait by *Y. enterocolitica* to titrate out host regulators responsible for actin polymerisation ^44^. YopH is multi-functional and disrupts pathways involved in both innate and adaptive immunity and is essential for the virulence of *Y. pestis*, *Y. pseudotuberculosis*, and *Y. enterocolitica* in mice ^45– 47^. YopH inhibits autophagy following binding of invasin or YadA (see next section) to β1-integrins ^48^ and also blocks phagocytosis in macrophages ^49, 50^ by dephosphorylating focal adhesion complex proteins, which disrupts the link to the actin cytoskeleton ^51, 52^.

The remaining two effectors (YopJ and YopM) down-regulate elements of the immune system, such as inflammation and leukocyte recruitment ^53– 57^. YopJ (YopP in *Y. enterocolitica*) is a serine/threonine/lysine acetyltransferase that catalyses the acylation of kinases, inhibiting their ability to activate the release of NF-Κβ, which would otherwise induce pro-inflammatory cytokine production ^58– 62^. Recently, YopJ was also shown to play an important role in inhibiting caspase-1 in activated macrophages ^63^.

YopM is translocated into macrophages ^64^ and may also be able to self-deliver into some human cells ^65^, yet it has no known enzymatic activity ^66^ and its true function has yet to be elucidated. Inside eukaryotic cells, YopM may interact with and stimulate cellular kinases ^67^ and is thought to localise to the nucleus ^68– 70^, where it may influence the expression of a range of genes, down-regulating many pro-inflammatory cytokines ^65, 71^, counteract the innate immune system by promoting depletion of natural killer cells in the liver, spleen, and blood ^72^, and also prevent pyroptosis by binding to caspase-1, inhibiting its activity ^73, 74^.

## Yersinia surface adhesins

For yersiniae to efficiently deliver Yops into the host, it is essential that they adhere to the host cell surface and remain in close association during the delivery process. To ensure that this is possible, the yersiniae produce virulence factors in addition to the T3SS. An active T3SS can deliver effector proteins into the host cell cytosol only if the bacterial cells make direct contact with, and bind tightly to, the host cell surface. Over the last 30 years, several chromosomally or plasmid-encoded protein virulence factors have been identified that play a variety of roles in host cell attachment prior to effector protein injection. In each case, attachment is not their exclusive function and not all are present or active in all three of the human pathogens. However, a combination of these proteins confers the ability to adhere to and invade host cells or bind sufficiently to ensure successful T3SS delivery of Yops.

## Invasin

**Figure 3.**
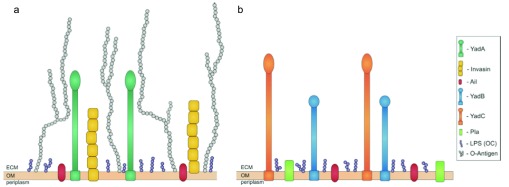
Virulence factors found on the surface of *Yersinia pseudotuberculosis*, *Yersinia enterocolitica* ( **a**), and *Yersinia pestis* ( **b**) Ail, YadB, and YadC are shared by all three pathogens – YadB and YadC are absent from panel (a) for clarity – while Pla is unique to *Y. pestis*. YadA and invasin are important adhesins in *Y. pseudotuberculosis* and *Y. enterocolitica* but are not expressed by *Y. pestis*. Reproduced from reference 95.

Invasin is a chromosomally encoded protein that mediates attachment to and entry into host cells by *Y. pseudotuberculosis* and *Y. enterocolitica* ^75^, although in *Y. pestis* it is a pseudogene and therefore inactive ^76^ ( Figure 3). Invasin promotes small intestine epithelial cell internalisation by binding to host cell target receptors known as β1-integrins ^77^ that present on the host cell surface. Integrins form clusters upon invasin binding, and the result is the rearrangement of the host cell cytoskeleton. This promotes phagocytosis and ultimately internalisation of the bacteria into the epithelial cells. In fact, invasin has a significantly greater (up to 100 times) affinity for some integrins than its natural ligand, fibronectin ^78^, and such strong associations are believed to be major contributing factors to the efficiency of internalisation and Yop delivery into host cells.

Invasin expression is regulated by both temperature and pH in *Y. enterocolitica* ^79, 80^. The invasin gene is maximally expressed at 26°C, peaking during late exponential/early stationary phase with lower expression levels observed at 37°C. This apparent contradiction, since invasin is required for infection at 37°C, was resolved when Pepe *et al*. revealed that the expression of invasin at 37°C was restored to levels seen at 26°C when the pH was reduced to 5.5. It has been suggested that rather than an experimental artefact, the expression of invasin at ambient temperatures could prepare the bacteria for infection following ingestion and promote rapid transcytosis through the epithelia ^81, 82^. The pH effect is not evident in *Y. pseudotuberculosis*, suggesting that the mechanisms of regulation of invasin expression may differ between the two species ^83^. Two regulators have been found to be important for invasin expression: RovA, required for the positive regulation of invasin, and YmoA, required for negative regulation ^83– 85^. Both RovA and YmoA recognise the promoter region of invasin and compete for binding. Once RovA is bound, it appears to prevent YmoA from binding, thus inhibiting negative regulation of invasin ^86, 87^. The expression of rovA is itself regulated by temperature via RovM, which acts as a repressor of rovA expression under inducing growth conditions ^88^.

## YadA

After crossing the intestinal epithelium, the major adhesin responsible for Yersinia contact with cells of the submucosa is the virulence plasmid-encoded protein YadA (recently reviewed by Mühlenkamp *et al*. ^89^) ( Figure 3). YadA expression is induced at or above 37°C ^90, 91^, and under these conditions it is so abundant that it can virtually coat the entire outer surface of the bacterial cell ^92^. Interestingly, despite YadA’s utility and abundance, *Y. pestis* possesses an inactive yadA pseudogene due to a single nucleotide deletion that results in a frame-shift mutation ^93^ ( Figure 3). Although *Y. pestis* does not produce a functional YadA protein, the chromosome carries two orthologues, YadB and YadC. Also found in *Y. pseudotuberculosis*, these two proteins are not thought to play a role in adherence but may contribute to host cell invasion. They may also be required for full virulence and lethality in bubonic but not pneumonic plague in mouse infection models ^94^.

YadA is a non-fimbrial adhesin ^95^ belonging to the trimeric autotransporter adhesin family members, which are usually referred to as obligate homotrimeric proteins. The protein is shaped like a lollipop, with an N-terminal globular head domain connected by a coiled-coil stalk to a C-terminal anchor domain embedded in the outer membrane ^92^. YadA has multiple functions but as an adhesin may act as a docking system, allowing the injectisome of the T3SS to come into contact with the target cell membrane to deliver the Yop effector proteins ^96^.

Until recently, it was thought that YadA bound only to the large proteins of the extracellular matrix – collagen, fibronectin, and laminin – which in turn bind β1-integrins ^97– 99^. However, Keller *et al*. ^100^ recently discovered that YadA-mediated adhesion may be facilitated by a broad range of host cell receptors and in the absence of β1-integrins may facilitate Yop injection via αV integrins as well as other unidentified cofactors. *Y. enterocolitica* YadA also binds leukocytes in a β1-integrin-independent manner during systemic infection ^101^, all of which suggests that YadA has the potential to target a broad range of cell types to ensure efficient Yop delivery.

The collagen-binding activity of YadA in *Y. enterocolitica* is an absolute requirement for pathogenicity; however, YadA is not essential for virulence in *Y. pseudotuberculosis* ^97^. YadA mediates adhesion to a number of cell types, including epithelial cells and macrophages, and can act as a haemagglutinin ^97^. In *Y. pseudotuberculosis*, YadA promotes the invasion of epithelial cells and is interchangeable with the activity of invasin ^102^, although *Y. enterocolitica* YadA is not as efficient an invasin as that of *Y. pseudotuberculosis* ^103^. YadA also mediates bacteria-bacteria autoagglutination, since the head domain has an affinity for itself ^92^. This self-affinity also promotes the formation of densely packed microcolonies that may promote antiphagocytic activity in *Y. enterocolitica*. YadA also binds to intestinal mucus ^104^ and plays a major role in conferring serum resistance ^105– 107^.

## Ail

The ail locus is chromosomally located and encodes a 17 kDa surface-associated protein ( Figure 3) that is thermally regulated, being maximally expressed at 37°C ^108, 109^. In *Y. enterocolitica*, Ail-directed adhesion to host cells shows more specificity than invasin, as it allows invasion of some cell lines, such as human laryngeal epithelial type 2 (HEp-2), human endometrial (HEC-1B), and Chinese hamster ovary (CHO) cells, but no invasion of Madin-Darby canine kidney (MDCK) cells ^110^. Both laminin and fibronectin are known targets for *Y. pestis* Ail binding ^111^ and vitronectin is actively recruited to the *Y. pestis* surface through the activities of Ail ^112^. Interestingly, *Y. pseudotuberculosis* Ail is unable to promote the attachment and invasion phenotypes when expressed in *Escherichia coli* ^113^. As with invasin, Ail-mediated tight attachment to host cells presumably ensures that Yop delivery is rapid and efficient ^114^. Aside from its adhesive properties, Ail also confers resistance to serum killing ^115^ in all three human pathogenic yersiniae. It is apparent that Ail plays a more prominent role in the virulence of *Y. pestis*, which is presumably owing to the fact that the other prominent virulence factors contributing many of the Ail functions in *Y. pseudotuberculosis* and *Y. enterocolitica* are dysfunctional.

## Psa – the pH6 antigen

The chromosomally encoded pH6 antigen (Psa) was originally identified in *Y. pestis* as a surface antigen expressed at mammalian body temperatures at pH values similar to those found in phagolysosomes ^116^. It was subsequently found to cause the agglutination of erythrocytes ^113, 117^. Further investigation revealed a cell surface complex composed of aggregates of a 15 kDa protein (PsaA) that requires two regulators, PsaE and PsaF, for maximal induction ^118, 119^. PsaA possesses a flexible fimbrial structure that is highly expressed during macrophage infection ^120^. Biochemical examination of Psa reveals that it binds to β1-linked galactosyl residues in glycosphingolipids ^121^, mainly of the type found in apolipoprotein-B-containing lipoproteins in human plasma, such as low-density lipoprotein (LDL) and in lipid rafts in macrophage membranes ^122^. Furthermore, Psa acts as a bacterial Fc receptor, binding human immunoglobulin (IgG) but not reacting with rabbit, mouse, or sheep IgG ^123^. As with the other adhesins, the activities of Psa appear to mediate Yop secretion. *Y. pseudotuberculosis* and *Y. enterocolitica* both produce a surface protein analogous to Psa but it is referred to as MyfA. Both Psa and MyfA coat the bacterial surface with a fibrillar matrix ^120, 124^ and in *Y. pseudotuberculosis* MyfA promotes attachment to tissue culture cells and haemagglutination ^113^.

## *Y. pestis* plasmid-specific virulence factors

Apart from the T3SS virulence plasmid, two other plasmids, pPCP and pMT (sometimes referred to as pFra) that are unique to *Y. pestis*, possess additional virulence factors. pPCP encodes the plasminogen activator Pla protease/adhesin ( Figure 3). Pla converts plasminogen to plasmin ^125, 126^, which then degrades extracellular matrices and confers on *Y. pestis* the ability to rapidly invade the host and migrate to lymphatic tissues ^127, 128^. The over-activation of plasmin results in laminin and fibrin clot degradation, exacerbating migration across host barriers ^129^, which is further compounded by the activities of Pla as an adhesin and an invasin ^130, 131^. pMT is responsible for the production of a murine toxin that is required during the colonisation of fleas ^132^.

## Concluding remarks

Over the last three decades, a considerable amount of detailed knowledge has accumulated that has enabled us to understand how the yersiniae colonise tissues and combat host defences during infection. While the Yersinia T3SS is perhaps the best understood system of its kind, many questions remain unanswered. For example, fully elucidating the function of YopM will offer an important step change, as will understanding more clearly the global molecular mechanisms that underpin the regulatory relationships that must exist between the T3SS system and the adhesins. It is also important to try to understand the relationships that exist between the different adhesins, how they compensate for each other, and which environmental signals dictate their site-specific expression. Finally, although the structures of many of the adhesins have been elucidated, there is certainly a need to better understand how they interact with different host ligands. While significant progress has been made in defining this sophisticated and finely tuned arsenal of virulence determinants, much more work is required to fully appreciate the success of the yersiniae as pathogens.
